# Supplementary material for: Optical framed knots as information carriers
Source: Nat Commun. 2020 Oct 9;11:5119. doi: 10.1038/s41467-020-18792-z (PMC7547656; doi:10.1038/s41467-020-18792-z)
Supplement: Supplementary file 1 — Supplementary Information [file 41467_2020_18792_MOESM1_ESM.pdf]

Supplementary Information for: Optical Framed Knots as Information Carriers

Larocque et al.

SUPPLEMENTARY FIGURES

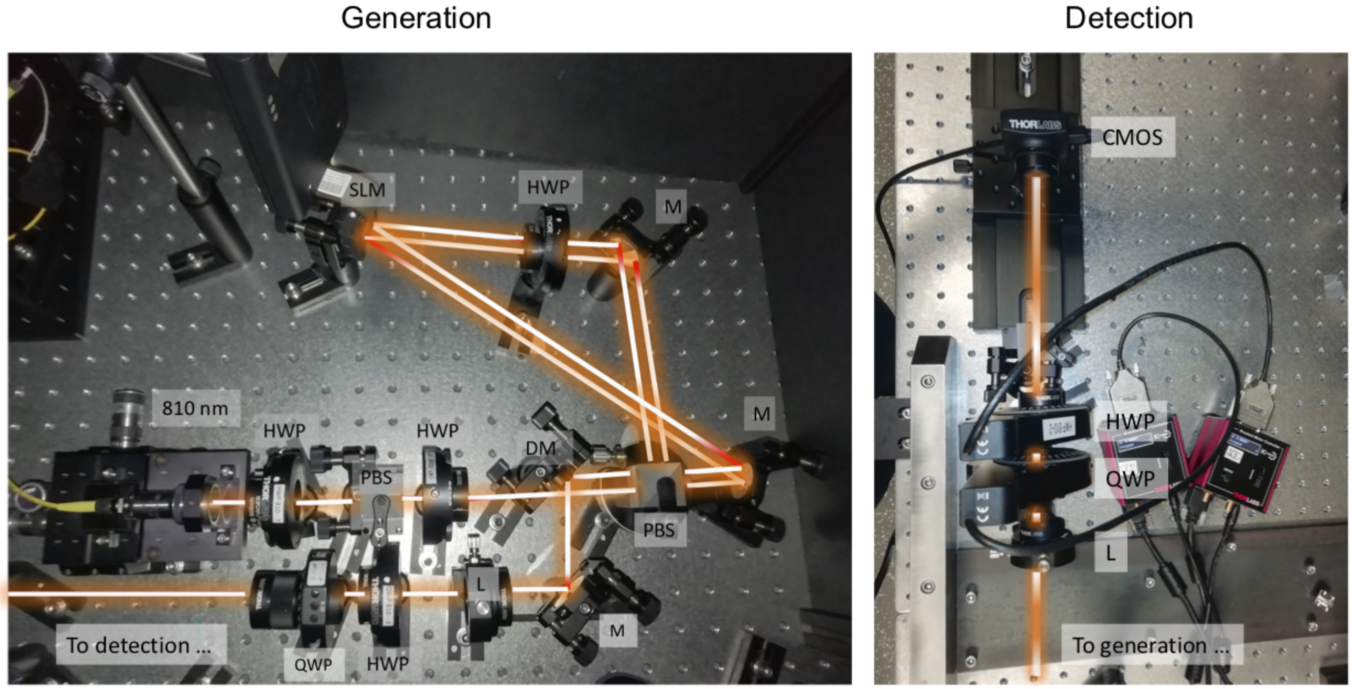

Supplementary Figure 1. **Photo of the experimental setup.** Left: Knot generation component of the setup. Right: Knot detection component of the setup. Figure legend: HWP: half-wave plate, PBS: polarizing beam splitter, M: mirror, SLM: spatial light modulator, DM: D-shaped mirror, L: lens, QWP: quarter-wave plate, CMOS: complementary metal–oxide–semiconductor camera.

SUPPLEMENTARY NOTE 1: POLYNOMIAL REPRESENTATION OF NON-PARAXIAL KNOTS

Here, we provide the formulation of the non-paraxial trefoil knotted ribbon displayed in Fig. 1 of the main text. To do so, we follow the derivation of the non-paraxial trefoil knot performed in [1]. Assuming that the electric field is circularly polarized ($\mathbf{e}_- = (\mathbf{x} - i\mathbf{y})/\sqrt{2}$), one can start this derivation with the knotted paraxial field [2]

$$E_-^{\mathrm{par}} = (1 - R^2 - R^4 + R^6 - 8e^{3i\phi}R^3) - 2iZ - 8iR^2Z + 18iR^4Z + 8Z^2 - 72R^2Z^2 - 48iZ^3, \quad (1)$$

where $R = krs^{-1}$ and $Z = kzs^{-2}$, (r, ϕ, z) are the cylindrical coordinates, k is the wave vector, and s is a scaling parameter. An $s \gg 1$ value corresponds to the paraxial regime, whereas an $s \approx 1$ entails non-paraxial behavior. Gauss' law determines the z component of the paraxial field, which is given by

$$E_z^{\mathrm{par}} = -\sqrt{2}is^{-1}Re^{-i\phi}[1 + 2R^2 - 3R^4 + 24Re^{3i\phi} + 4iZ(2 - 9R^2) + 72Z^2] + O(s^{-2}), \quad (2)$$

where higher orders in s^{-1} are ignored given that $s \gg 1$ for paraxial waves. One can then use Supplementary Equation 1 in conjunction with Bessel polynomials [3] to determine a generalized formulation of the trefoil knot accounting for non paraxial behavior. Doing so yields the field $\mathbf{E}^{\mathrm{k}} = E_-^{\mathrm{k}}\,\mathbf{e}_- + E_z^{\mathrm{k}}\,\mathbf{z}$, where

$$E_-^{\mathrm{k}} = E_-^{\mathrm{par}} + s^{-2}[8i(1 + 18s^{-2} - 9R^2)Z + 144Z^2]. \quad (3)$$

Gauss' law is then also used to derive the z component of the generalized field, which yields,

$$E_z^{\mathrm{k}} = E_z^{\mathrm{par}} + 4\sqrt{2}iRe^{-i\phi}s^{-3}[2 + 54s^{-2} - 9R^2 - 54iZ], \quad (4)$$

where all orders of s are now included. The knot considered in Fig. 1 of the main text is defined by $s = 4.5$. Note that these components are expressed in terms of polynomial beams [3]. Though they do consist of full solutions to Maxwell's equations,

they carry an infinite amount of energy and are thus not deemed physical. Nonetheless, the dynamics of these beams can still hold in physically realizable systems provided that the extent of the field enclosing the beam's vortices is large enough [1, 2]. More specifically, the diffraction length of the enclosing field, which could be a Gaussian beam for instance, must exceed the length over which the knot is formed in the polynomial beam.

We then proceed by coherently adding a left-circular polarized plane wave, to the above non-paraxial field. Given the polynomial nature of our analysis, we choose to represent this additional component as a complex constant. This component is represented by the E_+^{p} structure shown in Fig. 1c of the main text. This results in the formation of a complex vector field $\mathbf{E}(\mathbf{r}) = (E_x(\mathbf{r}), E_y(\mathbf{r}), E_z(\mathbf{r}))$ which can be expressed as [4, 5]:

$$\mathbf{E} = |\mathbf{E}|\,(\mathbf{A} + i\mathbf{B})\exp(i\epsilon), \tag{5}$$

where $|\mathbf{E}|$ is the amplitude of the field, $\mathbf{e} = \mathbf{A} + i\mathbf{B}$ is a complex vector representing the polarization ellipse traced by the electric field, and ϵ is a phase that is set such that $\mathbf{A}$ and $\mathbf{B}$ represent the major and minor axes of the polarization ellipse, respectively. Note that the scalar $\mathbf{e} \cdot \mathbf{e}$ represents the eccentricity of the polarization ellipse and is minimized for circular polarization where $\mathbf{e} \cdot \mathbf{e} = 0$ [5]. Furthermore, the orientation of $\mathbf{A} \times \mathbf{B}$ provides the vector normal to the oscillation plane of the polarization ellipse. We use these two quantities to reconstruct the frame of the C-line of the fields in Fig. 1 of the main text by first localizing the field's knotted C-line through regions where $\mathbf{e} \cdot \mathbf{e} = 0$. The results of this process are depicted in Fig. 1d of the main text. We then find the tangent and the $\mathbf{A} \times \mathbf{B}$ vector along this C-line. As discussed in the main text, taking the cross product of these two quantities allows us to obtain the knot's frame. Examples of the frames constructed from this method are shown in Fig. 1e of the main text. At this point, a few remarks can be made regarding the interplay between the knotted field $\mathbf{E}^{\mathrm{k}}$ and the plane wave E_+^{p}. On its own, $\mathbf{E}^{\mathrm{k}}$ has C-lines located by default along the nulls of the E_z field. The coherent addition of E_+^{p} deforms the C-line into a knot within the proximity of the one formed by E_-^{k}. When the amplitude of this additional field is increased, the knotted C-line gradually adopts the shape of the E_-^{k} knot and its frame experiences less influence from E_z^{k}. The formation of the C-lines in $\mathbf{E}_{\mathrm{k}}$ and how they are deformed by the addition of E_+^{p} are depicted in Figs. 1c,d of the main text, respectively. The normal of the circular polarization vector then gradually aligns itself to the beam's direction of propagation, z, thereby reducing the presence of z components in the C-line's framing illustrated in Fig. 1e of the main text. Note that expressing the amplitude of the field of E_+^{p} relative to $\mathbf{E}^{\mathrm{k}}$ becomes an ill-defined task because the depicted fields are formed with polynomial beams [3], which are not normalizable. We therefore abstain from relatively expressing the amplitude of E_+^{p} by simply expressing both fields as polynomials.

The sudden transition observed between the topologies of the C-lines formed with $E_+^{\mathrm{p}} = 0$ and $E_+^{\mathrm{p}} = 5$ further elucidates the importance of E_+^{p} in our framed knots' construction. Given that a knotted C-line formed with $\left|E_+^{\mathrm{p}}\right| = 0$ is entirely determined by the topology of E_z^{k} whereas that formed with $\left|E_+^{\mathrm{p}}\right| \gg 0$ is entirely determined by the topology of E_-^{k}, then there exists a range of $\left|E_+^{\mathrm{p}}\right|$ where the topology of the knotted C-line becomes unstable as it transitions from one form to the other. Note that such transitions are of little practical importance for structures realized within paraxial fields given the negligible presence of the z-polarized field.

SUPPLEMENTARY NOTE 2: LOW DIMENSIONAL TOPOLOGY

Combinatorial knot theory studies knots as planar diagrams instead of as embedded objects in 3–space. These diagrams are decomposed into tangles [6]. Knots and tangles are modified by *local moves*, which replace one tangle within a knot by another. Knots are thus revealed to be algebraic objects arising as concatenations of crossings (which are very simple tangles) in the plane [7].

The combinatorial paradigm of knot theory manifests a new philosophy of what constitutes algebra. For the combinatorial knot theorist, algebra no longer consists merely of formal manipulations of strings of symbols, but rather of operations and local modifications of labeled figures in the plane and in higher dimensions. This philosophy of diagrammatic algebra has become particularly well established in the representation theory of quantum groups, in higher category theory, and in quantum field theory [8–11].

Two planar diagrams are considered equivalent if one may be transformed into the other through a series of local deformations known as the Reidemeister moves, referring to the three kinds of archetypal manipulations shown in Supplementary Figure 2,

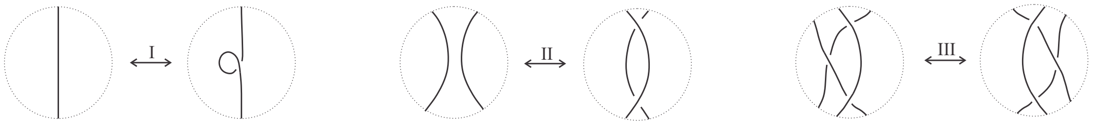

Supplementary Figure 2. **Diagrammatic representations of the three Reidemeister moves.**

In this work we chiefly care about framed knots and more broadly knotted ribbons (see Supplementary Figure 3). Mathematically speaking, they are embeddings of the solid torus in 3-sphere.

Definition 1 *A framed knot* (K, V) *in* S^3 *is a knot* K*, i.e., an embedding of* S^1*, equipped with a vector field* V *called a framing.*

The framing is characterized by a number, the *framing integer*, which is the linking number of the image of the ribbon $I \times S^1$ with the knot. In other words, it counts the number of times the vector field twists (2π rotations) around the knot. Knotted ribbons generalize framed knots to an odd number of half-twists, e.g., knotted Möbius bands.

The planar diagrams of knotted ribbons are respectively equipped with a (blackboard) framing representing the number of half-twists along the ribbon. The Reidemeister type-I move clearly changes the framing while the two other moves preserve it. For that reason, two knotted ribbons are considered equivalent if one may be transformed into the other by a sequence of type-II and type-III moves only, though it is worthwhile noting that a modified type-I move may be introduced which preserves the framing.

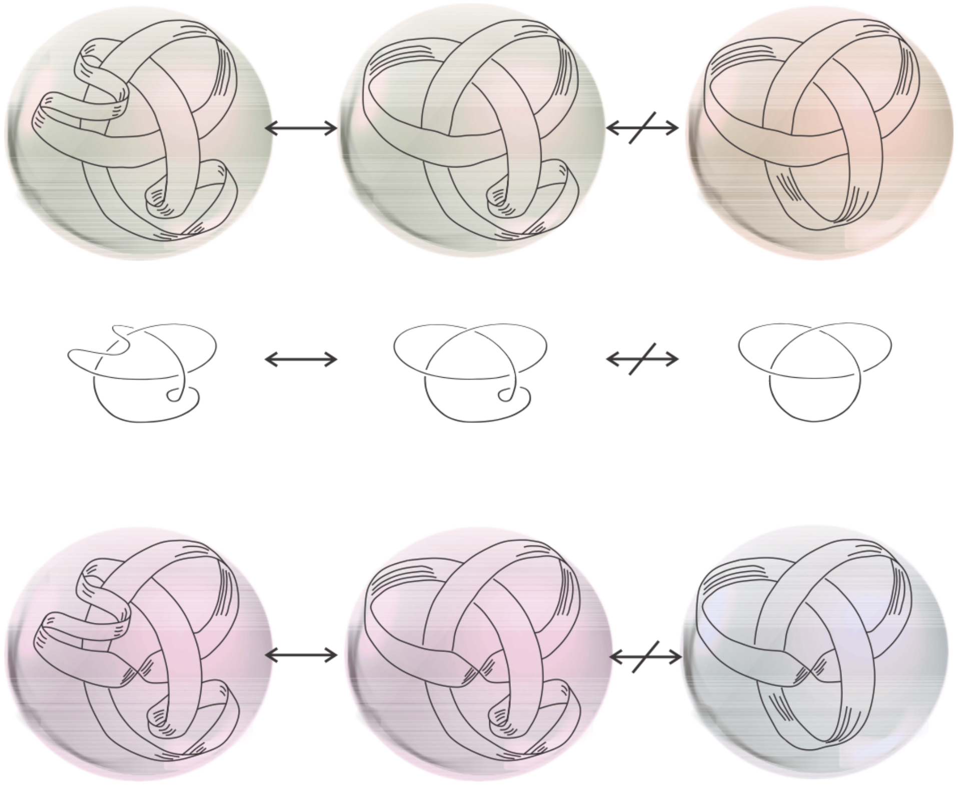

Supplementary Figure 3. **Equivalence of framed knots and ribbons.** The two leftmost framed trefoil knots in the upper row are equivalent for they are related by a Reidemeister type-II move. To their right is another, though not equivalent, framed trefoil (it lacks the full twist, the Reidemeister-I deformation, shared by the two other). The corresponding planar diagrams (excluding blackboard framing) are shown in the middle row. The bottom row illustrates three knotted ribbons – they all have an odd number (3) of half-twists. The two leftmost knotted ribbons are related by a Reidemeister type-II move whereas the one on the right is not equivalent to any of them (for it lacks the half-twist).

Braids are yet another family of tangles which, much like framed knots, are invariant to type-II and type-III moves. For a matter of fact, braids are purely algebraic entities defined via fundamental groups of a configuration space. The braid group on n strands, $\mathcal{B}_n$, consists of $n-1$ generators (represented as crossings between two nearby strands) and relations encoding the possible type-III moves. The type-II move is manifested by concatenating generators with their inverses.

Every knot may be represented as the closure of some braid (due to Alexander's theorem), namely, by attaching pairs of corresponding string ends in the braid. Similarly, every framed knot may be represented as the closure of some framed braid.

SUPPLEMENTARY NOTE 3: KNOTTED RIBBONS AND FRAMED KNOTS AS INFORMATION CARRIERS

As carriers of information, knotted objects may exhibit greater resilience to environmental disturbances than conventional data structures. The information content – the myriad of topological invariants – is generally insensitive to many of the deformations occurring when topological objects are materialized and handled. This section describes an approach, reminiscent of integer

factorization, that allows representing a number by a knotted ribbon or a framed knot in such as a way that its respective framed braid representation corresponds to the prime factorization of that number.

A. The framed braid group and its representation

We begin with the following observation concerning braid representations. As already pointed out, every unframed knot has a braid representation, i.e., a braid whose closure is that knot. The braid representation is not unique for there may be other braids whose closure is the same knot. These different braid representations are related by two types of local deformations known as Markov moves, i.e., the braid representation is in fact an equivalence class.

A framed knot, and more generally a closed knotted ribbon, likewise admits a framed braid representation, i.e., a framed braid whose closure is equivalent to the knotted ribbon. But in this case, we face an irreducible arbitrariness in the construction of the braid representation; there is more than one way to specify a framing for a braid from which a particular knotted ribbon is formed. Another way to see this is to imagine how half-twists are slided along the knotted ribbon such that every time it is cut into the same set of strands a different framed braid is formed.

Here we supplement the knotted ribbon with additional information, a pair of real numbers (α,β), that allows uniquely recovering its framed braid representation. The idea is analogous to prime factorization of integers; with the aid of (α,β) the knotted ribbon represents a natural number while the framed braid is akin to the (unique) prime factorization of this number.

A framed braid with n strands is a word in the group [12]

$$\mathcal{FB}_n = \left\langle \sigma_1,\ldots,\sigma_{n-1},\tau_1,\ldots,\tau_n \;\middle|\; \begin{array}{l} \sigma_{j+1}\sigma_j\sigma_{j+1} = \sigma_j\sigma_{j+1}\sigma_j, \\ \tau_i\tau_j = \tau_j\tau_i, \\ \tau_{j+1}\sigma_j = \sigma_j\tau_j,\ \sigma_j\tau_{j+1} = \tau_j\sigma_j \\ \sigma_i\sigma_j = \sigma_j\sigma_i,\ \tau_i\sigma_j = \sigma_j\tau_i \end{array} \right\rangle \tag{6}$$

where the second to last relation holds for $|i-j|>1$. The σ_j is the braiding generator of the jth and $(j+1)$th strands, and τ_j is the twisting generator of the jth strand. The generators and relations of the framed braid group are illustrated in Supplementary Figure 4.

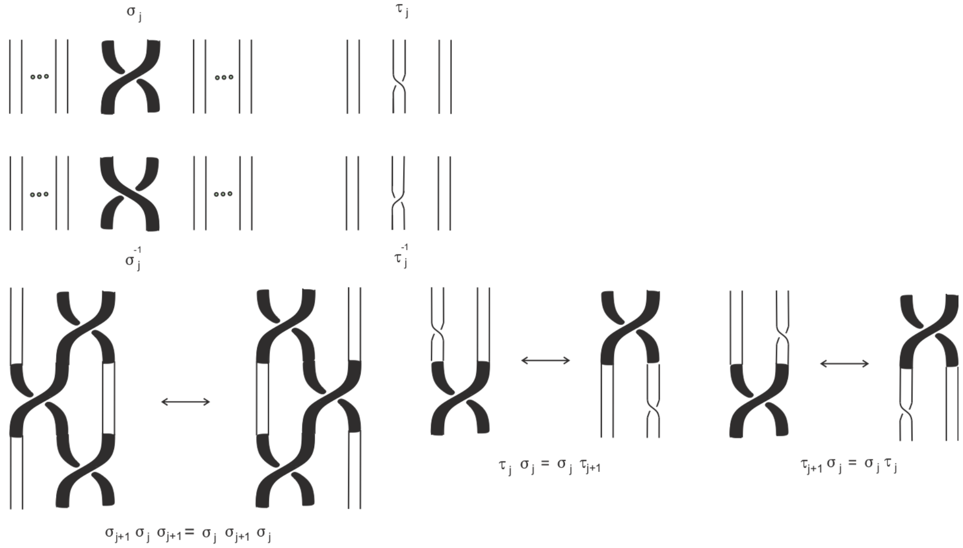

Supplementary Figure 4. Generators and relations of the framed braid group, $\mathcal{FB}_n$.

An unframed braid of n strands may act on the composite Hilbert space $\mathcal{H}^{\otimes n}$. The braid generators σ_j correspond to braiding operators,

$$R_j = \mathbb{1}^{\otimes(j-1)} \otimes R \otimes \mathbb{1}^{\otimes(n-j-1)} \tag{7}$$

where $\mathbb{1}$ is the identity operator, and R, a unitary operator on $\mathcal{H}\otimes\mathcal{H}$ that satisfies the Yang-Baxter equation,

$$(R\otimes\mathbb{1})(\mathbb{1}\otimes R)(R\otimes\mathbb{1}) = (\mathbb{1}\otimes R)(R\otimes\mathbb{1})(\mathbb{1}\otimes R) \tag{8}$$

As was shown in [13], this R may be an entangling operator acting on the composite system, e.g., $R|00\rangle = (|00\rangle + |11\rangle)\,/\,\sqrt{2}$.

A framed braid, $\mathcal{F}_n \times \mathcal{B}_n$, acts on the composite Hilbert space $\mathcal{H}^{\otimes n} \otimes \mathcal{H}^{\otimes n}$. This time the braiding operator R_j acts on the first (framing) space as a swap gate and as an entangling operator on the second (braid) space. In particular, it assumes the form

$$R_j = \left[\mathbb{1}^{\otimes(j-1)} \otimes \mathrm{SWAP} \otimes \mathbb{1}^{\otimes(n-j-1)}\right] \otimes \left[\mathbb{1}^{\otimes(j-1)} \otimes R \otimes \mathbb{1}^{\otimes(n-j-1)}\right] \tag{9}$$

The twisting operator corresponding to τ_j is the self-adjoint

$$T_j = \left(\sum_{k\neq j} |k\rangle\langle k| + \alpha|j\rangle\langle j|\right) \otimes \mathbb{1}^{\otimes n} \tag{10}$$

where the kets, $|k\rangle$, $k = 1, \ldots, n$, span the framing space, and α is some real positive number. It can be verified that R_j and T_j thus defined satisfy the relations of $\mathcal{FB}_n$.

B. Framed braids and prime factorization

The framed braid group admits a natural representation in Hilbert space. Because the two spaces (the framing and the braiding) do not interact, we may take the trace of each one of them separately. Consider a framed braid, a word w in the group $\mathcal{FB}_n$, whose representation is $A(w)$. The framing space of $A(w)$ is obtained by tracing over the braid space,

$$F(w) = \mathrm{Tr}_{\mathcal{B}}\left[A(w)\right] = \sum_{\{k|d_k\neq-\infty\}} \alpha^{d_k}|k\rangle\langle k| \tag{11}$$

where d_k is the number of half-twists along the kth strand exhibiting half-twists, i.e., $d_k = -\infty$ for untwisted strands. If we now assign a *distinct* prime p_k with each such strand while constraining α to positive integers, then the framed braid may be thought of as representing the prime factorization of some natural number encoded by the underlying framed knot. Here, we define this number as

$$N_{\alpha,\beta}(M) \overset{\mathrm{def}}{=} \beta^{(\alpha^M)} = \prod_{\{k|d_k\neq-\infty\}} p_k^{(\alpha^{d_k})} \tag{12}$$

where,

$$M = \log_\alpha \mathrm{Tr}\left[F(w)\log_\beta P\right] = \log_\alpha\left(\sum_{\{k|d_k\neq-\infty\}} \alpha^{d_k}\log_\beta p_k\right) \tag{13}$$

is the corresponding number of half-twists in the associated knotted ribbon (as every half twist, by definition, amounts to multiplication by α), and $P = \sum_k p_k|k\rangle\langle k|$ is the corresponding prime association matrix.

The number of half-twists in the braid representation amounts to that in the framed knot, $M = \sum_{\{k|d_k\neq-\infty\}} d_k$, which determines the logarithm basis as

$$\beta = \prod_{\{k|d_k\neq-\infty\}} p_k^{(\alpha^{d_k-M})} \tag{14}$$

The number $N_{\alpha,\beta}(M)$ is determined exclusively by the number of half-twists, M, in a the knotted ribbon and the pair (α,β). In other words, given α and β, this number is a topological invariant of the knotted ribbon. The prime factorization of $N_{\alpha,\beta}(M)$ encodes the number of half-twists per strand in the framed braid representation of the knotted ribbon. This encoding scheme is depicted in Supplementary Figure 5, which consists of the symbolic representation of Supplementary Equation 12.

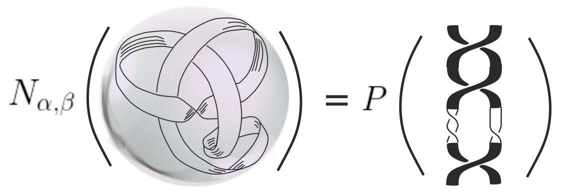

Supplementary Figure 5. Symbolic representation of the prime encoding of a framed braid. The prime factorization, P, of a framed braid corresponds to that of the integer $N_{\alpha,\beta}(M)$, which is determined by the pair (α,β) and the framed knot attributed to the braid.

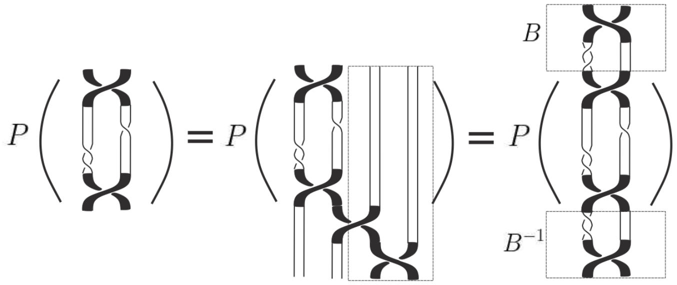

Supplementary Figure 6. **Markov moves attributed to framed braids.** The prime factorization of a framed braid (left) remains the same as the braid experiences stabilization (center) or conjugation (right).

As previously noted, the braid representation of a knot is not unique. Braid representations of the same knot are related by two kinds of Markov moves known as *stabilization* and *conjugation*. The same applies to knotted ribbons as long as the number of half-twists remains unchanged by these moves. As shown in Supplementary Figure 6, this invariance is carried over to the prime factorization captured by the framed braid. The left identity is stabilization – the act of adding untwisted strands, possibly interacting through Reidemeister-I moves, – which here amounts to padding the prime factorization with 1's, i.e., no half-twists are introduced by the newly added strands (primes). Note that there must be as much Reidemeister-I moves as their inverses for otherwise the number of half-twists would change. Similarly, conjugation of a braid A, i.e. the newly formed braid, BAB^{-1} on the right of Supplementary Figure 6, where $BB^{-1} = B^{-1}B = I$ is the framed unbraid, has the same number of half-twists as A, hence also the same prime factorization.

C. Some properties of $N_{\alpha,\beta}$

The number $N_{\alpha,\beta}(M)$ is a topological invariant of a knotted ribbon with M half-twists. Equivalent knotted ribbons necessarily have the same number $N_{\alpha,\beta}$ for a given pair (α,β). The converse clearly does not hold true; different knotted ribbons may be conceived having the same number $N_{\alpha,\beta}(M)$.

The role of $N_{\alpha,\beta}$ in the reconstruction of framed braids from knotted ribbons may be appreciated by considering the following scenario. Alice would like to send Bob a message which is here obtained as an output of a certain program running on some initial inputs, the set of numbers, d_k, $k = 1, 2, \ldots, n$. The program itself is a sequence of operations each taking as inputs some of these numbers or the outputs obtained by preceding operations. Running the program with the set of initial inputs is expected to yield Alice's message.

Alice conceives her program and its inputs as a framed braid. She identifies an operation with a sequence of crossings in the braid's planar diagram while the initial inputs are taken as the number of half-twists per strand. Alice has her program completely specified by the n-strand framed braid representation of her knotted ribbon K_A. To maintain some degree of privacy, she would like to send Bob her K_A rather than the original framed braid. She takes note of the fact that K_A may be complicated by a series of Reidemeister-II and III moves which would somewhat conceal the original framed braid. She proceeds by performing the following steps.

1. Choose a positive integer α.
2. Determine the framed braid representation of K_A; Allocate the number of half-twists in K_A to different strands of the braid, i.e., set d_k such that $M_A = \sum_k d_k$ is the total number of half-twists in K_A.
3. Assign prime numbers p_k to strands exhibiting half-twists.
4. Determine the number β according to Supplementary Equation 14.

Alice then sends Bob her knotted ribbon K_A and the pair of numbers α and β. Upon receiving these, Bob proceeds by computing $N_{\alpha,\beta}(M_A)$ whose prime factorization unfolds d_k. He can now recover an equivalent of the encoded framed braid from step (2) above.

SUPPLEMENTARY NOTE 4: DERIVATION OF THE PARAMETRIZED KNOTTED FIELD

We follow the method outlined in [14] to construct the knotted fields of the optical beams used in this work. We start by parametrizing the transverse coordinates of the braid representation of the trefoil and cinquefoil knots with respect to the height

coordinate $h \in [0, 2\pi]$. The Cartesian representation of these coordinates is given by:

$$X_j^r(h) = a\cos\left(\frac{1}{2}[rh + 2\pi(j-1)]\right), \qquad Y_j^r(h) = b\sin\left(\frac{1}{2}[rh + 2\pi(j-1)]\right), \tag{15}$$

where $i = \sqrt{-1}$, $j = 1, 2$ refers to one of the two strands in the braid, a and b are postive scaling constants, and $r = 3, 5$ for the trefoil and cinquefoil knots, respectively. We then express these coordinates in terms of $v = \exp(ih)$, thereby yielding

$$X_j^r(v) = \frac{a}{2}\left(v^{r/2}e^{i\pi(j-1)} + v^{*r/2}e^{-i\pi(j-1)}\right), \qquad Y_j^r(v) = \frac{b}{2i}\left(v^{r/2}e^{i\pi(j-1)} - v^{*r/2}e^{-i\pi(j-1)}\right), \tag{16}$$

where $v^* = \exp(-ih)$. We then express the braid as the roots of the following complex polynomial

$$p_v^r(u) = \prod_{j=1}^{2}(u - Z_j^r(v)) \tag{17}$$

where $Z_j^r(v) = X_j^r(v) + iY_j^r(v)$ and $u = x + iy$ is a complex value expressed in terms of the transverse Cartesian coordinates (x, y). We may then convert this polynomial into a complex field with knotted complex roots. This task is achieved with the stereographic projection

$$u = \frac{\rho^2 + z^2 - 1 + 2iz}{\rho^2 + z^2 + 1}, \qquad v = \frac{2\rho e^{i\varphi}}{\rho^2 + z^2 + 1}, \tag{18}$$

where (ρ, φ, z) refer to the cylindrical coordinates of the space in which the knot is embedded. Note that this projection effectively maps the h coordinate of the space in which the braid is embedded to the φ coordinate of the knot. For the cases of the trefoil ($r = 3$), and cinquefoil ($r = 5$) knots, the numerator of this expression at the $z = 0$ plane are respectively given by

$$p^{r=3}(\rho, \varphi, z = 0) = 1 - \rho^2 - 4(a^2 - b^2)\rho^3 - \rho^4 + \rho^6 - 2(a-b)^2\rho^3 e^{-3i\varphi} - 2(a+b)^2\rho^3 e^{3i\varphi}, \tag{19}$$

$$p^{r=5}(\rho, \varphi, z = 0) = 1 + \rho^2 - 2\rho^4 - 16(a^2 - b^2)\rho^5 - 2\rho^6 + \rho^8 + \rho^{10} - 8(a-b)^2\rho^5 e^{-5i\varphi} - 8(a+b)^2\rho^5 e^{5i\varphi}. \tag{20}$$

As demonstrated in Supplementary Figure 7a, reducing the b parameter of Eqs. (19,20) compresses the braid along the y direction. For the knot obtained from the projection of this braid, the observed compression is now along z, thereby resulting in a shorter knot. This corresponding compression is displayed in Supplementary Figure 7b,c. As further discussed in Supplementary Note 5, this compression property can be used to stabilize the frame of the knots generated in this work.

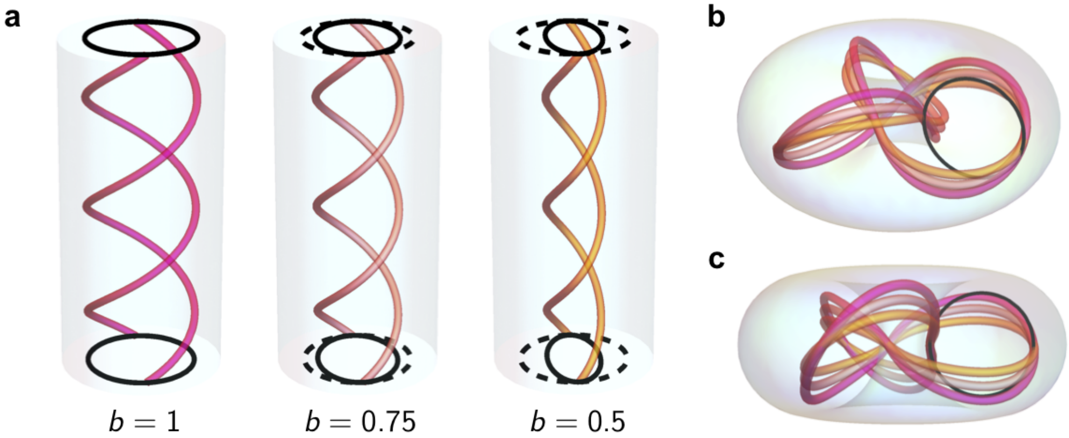

Supplementary Figure 7. **Effect of reducing the b parameter of the knot's parametrization. a,** Braids formed by the zeros of the complex polynomial defined in Supplementary Equation 17 with $r = 3$ and $a = 1$. The solid ellipses at the ends correspond to the ones traced out by the strands of the braids. The dashed ellipses are traced out by the braid with $b = 1$. **b,** Corresponding knots formed by the stereographic projection of the braids shown in (**a**) as prescribed by Supplementary Equation 18. **c,** Side view of the knot shown in (**b**) emphasizing the compression induced by a decreasing b parameter.

To obtain the optical fields $\psi_{a,b,s}^{\mathrm{Tref}}(\varrho, \varphi)$ and $\psi_{a,b,s}^{\mathrm{Cinq}}(\varrho, \varphi)$ provided in the main text, we take the projections from Eqs. (19,20), and let $\rho \mapsto \varrho$, where $\varrho = \rho/w_0$ is a dimensionless counterpart to the cylindrical radial coordinate ρ and w_0 refers to a scaling

factor. We then define the optical fields as the stereographic projections modulated by a Gaussian function, i.e. $\psi^r_{a,b,s}(\varrho,\varphi) = p^r(\varrho,\varphi)\,\exp\left(-(\varrho/s)^2/2\right)$, thereby resulting in the field formulations presented in the main text:

$$\psi^{\mathrm{Tref}}_{a,b,s}(\varrho,\varphi,z=0) = (1-\varrho^2-4(a^2-b^2)\varrho^3-\varrho^4+\varrho^6-2(a-b)^2\varrho^3e^{-3i\varphi}-2(a+b)^2\varrho^3e^{3i\varphi})\,e^{-(\varrho/s)^2/2}, \tag{21}$$

$$\psi^{\mathrm{Cinq}}_{a,b,s}(\varrho,\varphi,z=0) = (1+\varrho^2-2\varrho^4-16(a^2-b^2)\varrho^5-2\varrho^6+\varrho^8+\varrho^{10}-8(a-b)^2\varrho^5e^{-5i\varphi}-8(a+b)^2\varrho^5e^{5i\varphi})\,e^{-(\varrho/s)^2/2}. \tag{22}$$

SUPPLEMENTARY NOTE 5: RESILIENCE OF THE FRAME

The effect of reducing the b value of the knot's parametrization can also be seen in the optical knots presented in this work. To illustrate, we consider the propagation of space-varying polarized light beams that can be conveniently expressed as the coherent superposition of two components in the circular polarization basis. As shown in Fig. 3b of the main text, the left circular polarization term is defined by a large Gaussian beam. The right circular polarization component consists of the scalar fields shown in Supplementary Figure 8a. In the latter, we observe that the field's phase vortices become progressively asymmetric as b is decreased. This asymmetry causes the resulting polarization field of the beam to experience a contraction in the C-line forming a knotted structure. The contraction, displayed in the theoretical optical knots of Supplementary Figure 8b, is observed to be along the beam's longitudinal, z, direction. Furthermore, the motion of the C-line in the transverse plane, xy, becomes a lot more definitive. The knots formed by experimental structures with the same parameters are illustrated in Supplementary Figure 8c. Though not as pronounced as their theoretical counterparts, one can still observe some features that are indicative of the knot's longitudinal compression and the sharper transverse motion that is brought with the reduction of b.

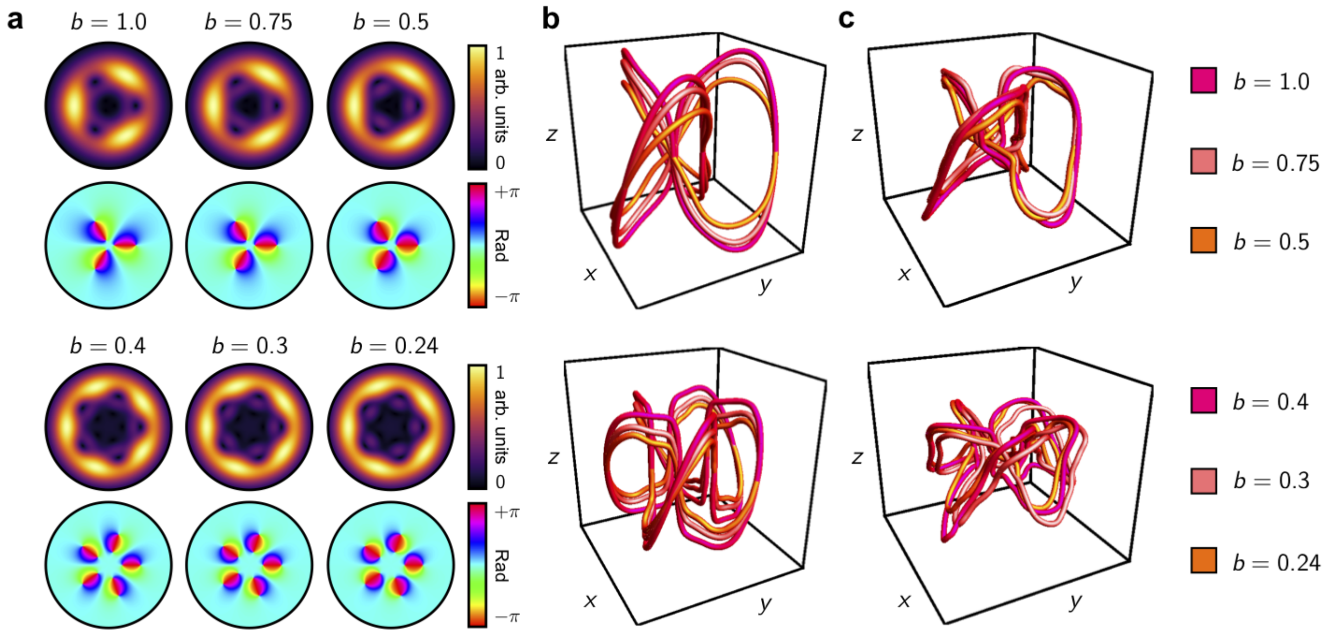

Supplementary Figure 8. **Effect on reducing the b parameter on the knotted optical field. a,** Amplitude and phase profiles of $\psi^{\mathrm{Tref}}_{a=1,b,s=1.2}$ (top) and $\psi^{\mathrm{Cinq}}_{a=0.5,b,s=0.65}$ (bottom). **b,** Knots expected from theory resulting from the paraxial propagation of the optical fields shown in (**a**). **c,** Knots obtained from experiment resulting from the holographic generation of optical beams based on the field profiles shown in (**a**).

One of the main sources of perturbation in the frame of the knots studied in this work are attributed to regions of the C-line that are very close to being parallel to the beam's direction of propagation. In these regions, all of the polarization vectors enclosing the C-line have a major axis that is close to being perpendicular to the knot's trajectory, and therefore to defining the knot's frame. Hence, any small perturbation in the C-line's trajectory, be it numerical or experimental, can have drastic effects on the knots frame and change its total number of half-twist. Therefore, having a knotted C-line with less longitudinal motion, such as those with lower b values, should help with improving the frame's resiliency to perturbations.

Another source of perturbation involves the birth of spurious C-lines that merge with those of the knot. Though they may not affect the knot's topology, they may introduce some swirls in the C-lines which affect the number of half-twists in the frame.

The reconstructed frame of these experimental structures is illustrated in Supplementary Figure 9. As mentioned earlier, decreasing the b parameter of the knotted field results in C-lines with sharper trajectories that are less prone to be oriented along

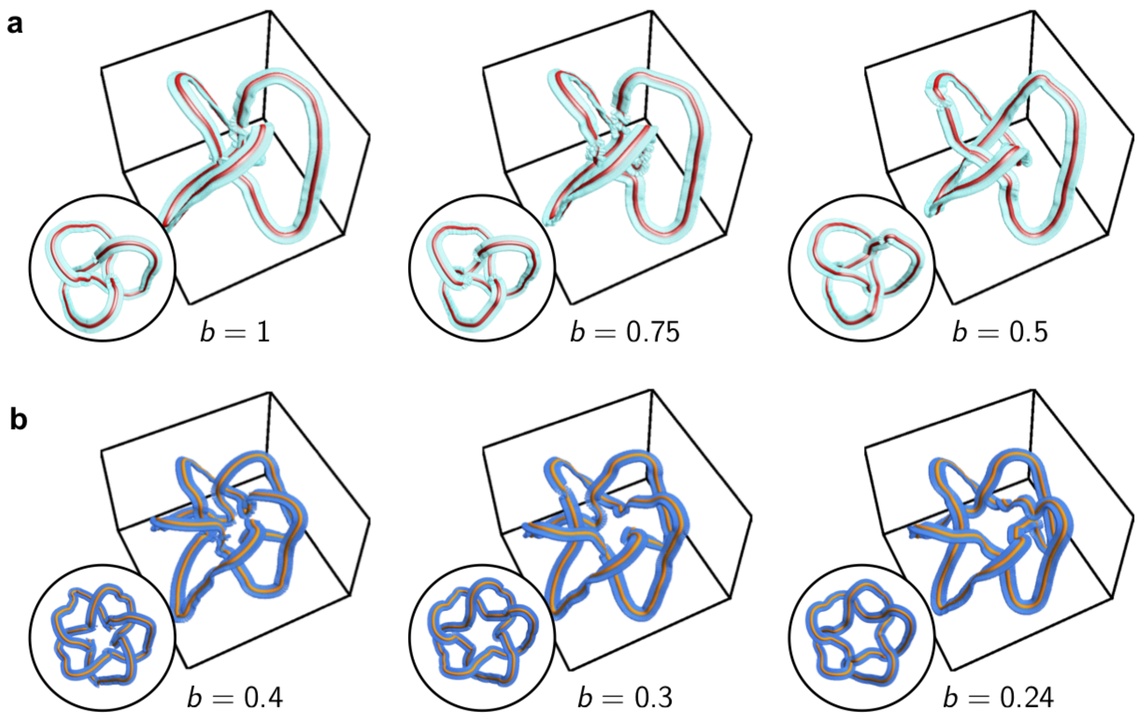

Supplementary Figure 9. **Frame stability of experimentally realized knotted C-lines. a,** Experimental framed trefoil knots defined by parameters of $s = 1.2$ and $a = 1$ and b values of 1, 0.75, and 0.5. **b,** Experimental framed cinquefoil knots defined by parameters of $s = 0.65$ and $a = 0.5$ and b values of 0.4, 0.3, and 0.24. Top views of the knots are provided as insets.

the beam's direction of propagation. Furthermore, fields with lower b parameters also tend to have less secondary singularities affecting the global structure of the knotted C-line. As a result, the frame of these structures globally appears to be more stable.

SUPPLEMENTARY NOTE 6: FRAMED KNOT EXTRACTION FROM RAW DATA

We rely on the procedure outlined in Supplementary Figure 10 to extract the framed knots formed by our optical structures. In the latter, we illustrate this process on the experimental trefoil knot presented in Fig. 4b of the main text. After recording polarization projections of the knotted C-line at several planes along the beam's propagation, we take these raw projections and make them go through a non-aggressive low-pass (LP) filter. This step is depicted in Supplementary Figure 10a. The filtering is performed in order to remove defects associated with dust and damage on the CMOS camera used to capture the image. In Fourier space, the filter specifically consists of a circular box function with a radius of 40 pixels. The filtered projections are then used to calculate the beam's reduced Stokes parameters, defined as

$$s_1 = (H - V)/I_0 \qquad s_2 = (A - D)/I_0 \qquad s_3 = (L - R)/I_0 \tag{23}$$

where H, V, A, D, L, and R represent the horizontal, vertical, anti-diagonal, diagonal, left circular, and right circular projections, respectively. I_0 represents the total intensity of the beam. The positions of the C-lines are then extracted by finding intersections of the phase contours formed by the field $s_1 + is_2$ [15]. This process is illustrated in Supplementary Figure 10b. We then proceed by connecting the location of the C-lines extracted at each measurement plane into the beam's underlying knot. In order to mitigate the effects related to the pixelation of the CMOS measurements, we make the transverse coordinates, i.e., x and y, of the resulting curve go through a Gaussian filter with a width of one bin. A spline going through each of the filtered points is then interpolated to obtain a continuous representation of the curve. The raw coordinates of the knotted C-line along with the spline arising from the interpolation of the filtered data are shown in Supplementary Figure 10c. The raw coordinates of the C-line along with its Gaussian filtered transverse coordinates are plotted in Supplementary Figure 10d. Once we have reconstructed the continuous representation of the knot, we scale it to fit within the proximity of the torus obtained from the stereographic projection of the cylinder enclosing the braid representation of our knot. An image of such a scaled knot along with said torus is provided in Supplementary Figure 10e. Finally, the frame of the knot is obtained by taking the cross product of the knot's tangent and the normal of the C-line's oscillation plane. For the case of paraxial structures, the normal is oriented along the

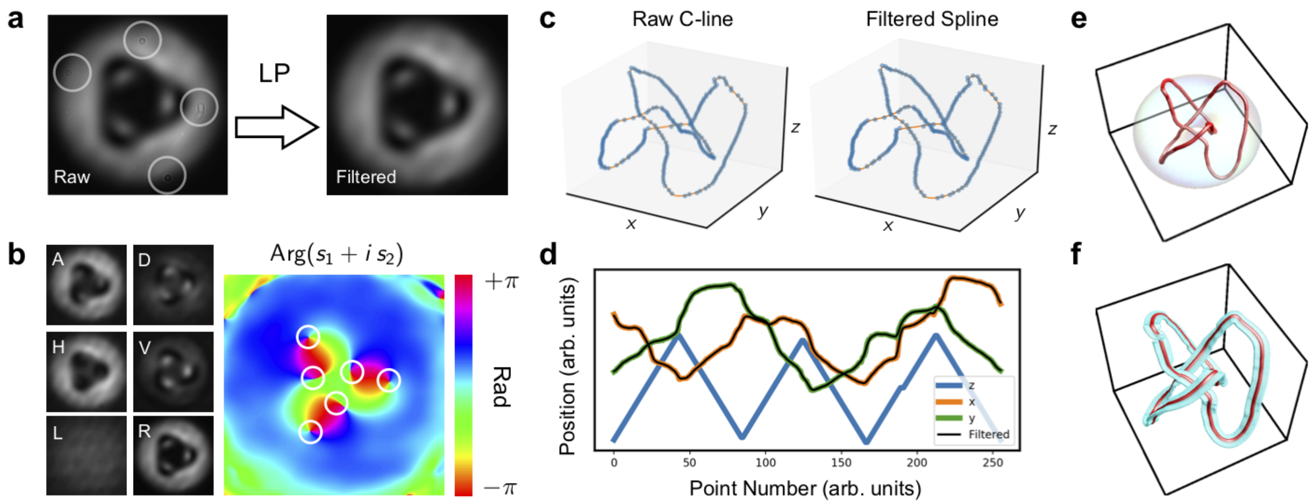

Supplementary Figure 10. **Reconstruction of optical framed knots from raw data. a,** Polarization projections are first low-pass (LP) filtered to remove any defects in the images. Examples of such defects are circled in the raw image. **b,** The filtered polarization projections are then used to obtain the Stokes parameters, s_i, of the beam at various planes along the beam's propagation. Intersections of the phase contours of the field formed by $s_1 + is_2$, herein labeled in the figure, may then pinpoint the location of the C-lines. **c,** Knots formed by the raw coordinates of the C-line extracted in (**b**) and by the spline interpolated C-line where the x and y coordinates have been subjected to a Gaussian filter. **d,** Coordinates of the curve formed by the C-line along with the Gaussian filtered transverse coordinates. **e,** Scaled version of the interpolated knot fitting within the torus related to the stereographic projection used to extract the knot's braid representation. **f,** Final knotted structure accompanied by the frame determined by the oscillation plane of the C-line.

beam's direction of propagation, i.e. the z axis, thereby confining the frame within the xy plane. An image of the resulting structure is displayed in Supplementary Figure 10f.

Supplementary References

[1] Sugic, D. & Dennis, M. R. Singular knot bundle in light. *J. Opt. Soc. Am. A* **35**, 1987–1999 (2018).
[2] Dennis, M. R., King, R. P., Jack, B., O'Holleran, K. & Padgett, M. J. Isolated optical vortex knots. *Nat. Phys.* **6**, 118–121 (2010).
[3] Dennis, M. R., Götte, J. B., King, R. P., Morgan, M. A. & Alonso, M. A. Paraxial and nonparaxial polynomial beams and the analytic approach to propagation. *Opt. Lett.* **36**, 4452–4454 (2011).
[4] Berry, M. V. & Dennis, M. R. Polarization singularities in isotropic random vector waves. *Proc. R. Soc. A* **457**, 141–155 (2001).
[5] Bliokh, K. Y., Alonso, M. A. & Dennis, M. R. Geometric phases in 2d and 3d polarized fields: geometrical, dynamical, and topological aspects. *Rep. Prog. Phys.* **82**, 122401 (2019).
[6] Conway, J. H. An enumeration of knots and links, and some of their algebraic properties. In *Computational Problems in Abstract Algebra*, 329–358 (1970).
[7] Jones, V. F. R. Planar algebras. Preprint at https://arxiv.org/abs/math/9909027 (1999).
[8] Fröhlich, J. & Kerler, T. *Quantum groups, quantum categories and quantum field theory* (Springer, 2006).
[9] Bilson-Thompson, S., Hackett, J., Kauffman, L. & Smolin, L. Particle identifications from symmetries of braided ribbon network invariants. Preprint at https://arxiv.org/abs/0804.0037 (2008).
[10] Bilson-Thompson, S., Hackett, J. & Kauffman, L. H. Particle topology, braids, and braided belts. *J. Math. Phys.* **50**, 113505 (2009).
[11] Gresnigt, N. Knotted boundaries and braid only form of braided belts Preprint at https://arxiv.org/abs/1808.03910 (2018).
[12] Ko, K. H. & Smolinsky, L. The framed braid group and 3-manifolds. *Proc. Am. Math. Soc* **115**, 541–551 (1992).
[13] Kauffman, L. *Knots and Physics* (World Scientific: Singapore, 1991).
[14] Bode, B., Dennis, M. R., Foster, D. & King, R. P. Knotted fields and explicit fibrations for lemniscate knots. *Proc. R. Soc. A* **473**, 20160829 (2017).
[15] Larocque, H. *et al.* Reconstructing the topology of optical polarization knots. *Nat. Phys.* **14**, 1079–1082 (2018).
